# Risk estimation and risk prediction using machine-learning methods

**DOI:** 10.1007/s00439-012-1194-y

**Published:** 2012-07-03

**Authors:** Jochen Kruppa, Andreas Ziegler, Inke R. König

**Affiliations:** Institut für Medizininsche Biometrie und Statistik, Universität zu Lübeck, Universitätsklinikum Schleswig-Holstein, Campus Lübeck, Maria-Goeppert-Str. 1, 23562 Lübeck, Germany

## Abstract

After an association between genetic variants and a phenotype has been established, further study goals comprise the classification of patients according to disease risk or the estimation of disease probability. To accomplish this, different statistical methods are required, and specifically machine-learning approaches may offer advantages over classical techniques. In this paper, we describe methods for the construction and evaluation of classification and probability estimation rules. We review the use of machine-learning approaches in this context and explain some of the machine-learning algorithms in detail. Finally, we illustrate the methodology through application to a genome-wide association analysis on rheumatoid arthritis.

**Electronic supplementary material:**

The online version of this article (doi:10.1007/s00439-012-1194-y) contains supplementary material, which is available to authorized users.

## Introduction

Unraveling the genetic background of human diseases serves a number of goals. One aim is to identify genes that modify the susceptibility to disease. In this context, we ask questions like: “Is this genetic variant more frequent in patients with the disease of interest than in unaffected controls?” or “Is the mean phenotype higher in carriers of this genetic variant than in non-carriers?” From the answers, we possibly learn about the pathogenesis of the disease, and we can identify possible targets for therapeutic interventions. Looking back at the past decade, it can be summarized that genome-wide association (GWA) studies have been useful in this endeavor (Hindorff et al. 2012).

Another goal is to classify patients according to their risk for disease, or to make risk predictions. For classification, also termed pattern recognition, typical questions are: “Is this person affected?”, which asks for a diagnosis, or “Will this individual be affected in a year from now?”, thus asking for a prognosis, or “Will this patient respond to the treatment?”, and “Will this patient have serious side effects from using the drug?” These questions ask for a prediction. In each case, a dichotomous yes/no decision has to be made.

In risk prediction, in contrast, we ask for probabilities such as “What is the probability that this individual is affected?”, or “What is the probability that this person will be affected in a year from now?”

These two concepts, classification and risk prediction, have received different levels of attention, and this by different groups. Specifically, classification is considered mainly using nonparametric approaches by the machine-learning community, while estimation of probabilities is generally approached by statisticians using parametric methods, such as the logistic regression model. Probability estimation at the subject level has a long-standing tradition in biostatistics, since it provides more detailed information than a simple yes/no answer, and applications include all areas of medicine (Malley et al. 2012). Since in the biostatistical community the term “risk prediction” is reserved for therapies, thus by calling for treatment response probabilities or side effects probabilities, we will avoid this term in the following and use the more general term of probability estimation (Steyerberg 2009).

It is important to emphasize that neither classification nor probability estimation automatically follow from association results. To put it more clearly, association means that the chance to be affected is, in the mean, greater in those carrying the disease genotype than in those who do not. However, when looking at the distributions of probabilities in cases and controls, there will often be a large overlap and the boundary between the two groups will not be sharp. Hence, the ability to discriminate cases from controls based on the genotype—the binary classification problem—is difficult.

When we consider classical measures for strength of association on the one hand, such as the odds ratio (OR), and for classification on the other hand, such as sensitivity (sens) and specificity (spec), there is a simple relationship between them with ${\text{OR}} = \frac{\text{sens}}{{1 - {\text{sens}}}} \cdot \frac{\text{spec}}{{1 - {\text{spec}}}}$ (Pepe et al. 2004). This relationship can be used to demonstrate that an single nucleotide polymorphism (SNP) can show a strong association but be a poor classifier. For example, if an SNP has a high sensitivity of 0.9 and a strong association of OR = 3.0, the specificity is only 0.25. Many more examples for this are given in the literature (Cook 2007; Wald et al. 1999). This result does not mean that either association studies or classification rules are not worthwhile. Instead, we should keep in mind that association, classification and probability estimation are different aims with their own values.

In the following, we will focus on classification and probability estimation based on GWA data. For this, we will describe in the next section how to construct and evaluate classification and probability estimation rules. In recent years, approaches from the machine-learning community have received more attention for this. Therefore, we will present a systematic literature review on the use of machine-learning methods. Some of these methods will then be described in more detail before we finally show examples of construction and evaluation of classification and probability estimation rules using a number of different methods on data from a GWA study on rheumatoid arthritis.

## Construction and evaluation of a classification/probability estimation rule

**Fig. 1:**
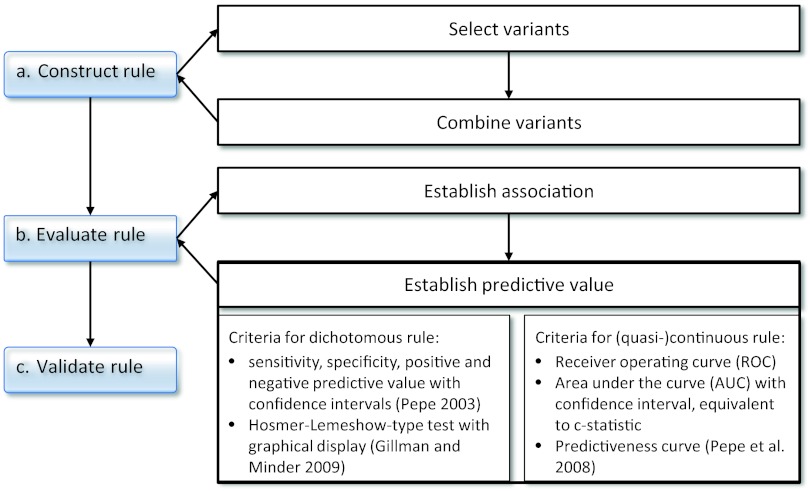
Path to construct, evaluate and validate a rule of classification or probability estimation

The overall process of rule construction and evaluation is shown in Fig. 1.

### How can a rule be constructed?

In the first step of rule construction (Fig. 1, part a), the variants to be used in the rule are selected, and this is in most cases based on the *p* values from association analyses of single marker analyses. In the simplest of all cases, the rule uses only the genotype of one SNP, and subjects are assigned a higher risk if they carry one (or two) susceptibility variant(s). Usually, however, a number of SNPs fulfilling some criterion are combined to a score. For the construction of the rule from the selected SNPs, a score is often used that simply counts the number of predisposing variants a single subject carries. This assumes that all variants contribute equally to the risk, and a more sophisticated rule weights the variants depending on their respective genetic effect (Carayol et al. 2010). Ideally, these genetic effects are estimated in a multivariate model, but often the results from single SNP analyses are used in most applications. It is also possible to select SNPs and construct the rule within the same analysis by using, e.g., penalized regression approaches (Kooperberg et al. 2010).

There has been a discussion about the number of SNPs to be integrated in a score. In many applications, SNPs were used that were genome-wide significant in previous analyses. As a result, typically less than 20 SNPs were combined. However, some examples have shown experimentally (Evans et al. 2009; Kooperberg et al. 2010; Wei et al. 2009) and theoretically (Zollanvari et al. 2011) that the results can not only be improved by using thousands of SNPs, but also require a high number of SNPs for good classification. In addition, a good prediction is often achieved more easily if established non-genetic clinical risk factors are incorporated into the model.

### How can a rule be evaluated? Using the ACCE model

Having constructed a rule, its performance needs to be evaluated in the second step (Fig. 1, part b). This evaluation requires additional approaches that can be illustrated using the framework of the ACCE project (Haddow and Palomaki 2004). Details on this project can be found on the Web site (http://www.cdc.gov/genomics/gtesting/ACCE/) as well as in chapter 14 of Ziegler and König (2010) and in Ziegler et al. (2012). Within this framework, we can evaluate predictive tests based on genetic variants that may or may not include non-genetic risk factors.

In brief, ACCE is an acronym for the following criteria used to evaluate predictive genetic tests: (A)nalytic validity evaluates how well the test is able to measure the respective genotypes. (C)linical validity is a criterion for how consistently and accurately the test detects and predicts the respective disease. (C)linical utility focuses on the influence of the test on outcome improvement for the patient, and (E)LSI comprises (E)thical, (L)egal and (S)ocial (I)mplications of the genetic test. Our aim here is the statistical evaluation of the classification and probability estimation rule, which is why we will focus on the clinical validity of the test.

For this, we firstly require established associations with the disease of interest. These are rendered from candidate gene association studies or from classical GWA studies and they need to be extensively validated (König 2011).

Secondly, as indicated above, the predictive value of the test needs to be established that indicates how well the test is able to differentiate between cases and controls and/or how good the probability estimates are. Specifically, the test needs to show calibration and discrimination. For a good calibration, the predicted probabilities agree well with the actual observed risk, i.e., the average predicted risk matches the proportion of subjects who actually develop the disease. Ideally, this should hold both for the overall study population and for all important subgroups. Reasonable measures for discrimination depend on the scale of the rule result. This might be dichotomous, because it is based on a single SNP only, or because the algorithm used for constructing the rule renders a binary classification. Alternatively, it might be (quasi-) continuous, as is the case if a score has been constructed, or if the algorithm renders risk probabilities. The respective measures are shown in Fig. 1, part b, right-hand side.

The classical measures of area under the curve (AUC) and* c*-statistic have often been criticized. For example, the* c*-statistic is not clinically meaningful, and a marginal increase in the AUC can still represent a substantial improvement of prediction at a specific important threshold (Pepe and Janes 2008). Also, the absolute risk values for individuals are not visible from this, and the AUC is not a function of the actual predicted probabilities (Pepe and Janes 2008). It has therefore been emphasized that the evaluation of the clinical validity should not rely on a single measure, but should be complemented by alternative approaches such as the predictiveness curve.

To evaluate predicted probabilities the Brier score (BS), which is given by the average over all squared differences between an observation and its predicted probability, is preferably used. The Brier score is a so-called proper score (Gneiting and Raftery 2007), it can be estimated if the probability is estimated consistently (Malley et al. 2012), and its variance can be estimated and used to construct confidence intervals (CIs) (Bradley et al. 2008).

If the genetic test is to be compared to a standard risk prediction tool, e.g., based on clinical parameters, measures can be used that are based on the re-classification of subjects as described in detail by Cook (2007) and Pencina et al. (2008).

It should be noted that there are no general thresholds that define a test to be clinically valid. For example, a model is not good in all cases where the AUC exceeds, say, 0.8. Alternative prediction models, the aim of testing, the burden and cost of disease, and the availability of treatment always need to be considered. Therefore, a detailed evaluation of the constructed models is necessary (Teutsch et al. 2009).

### How can validation of the rule be established?

The evaluation of a probability estimation or classification rule comprises the validation of its performance in further steps (Fig. 1, part c). Specifically, validation of a rule means that it acts accurately on new, independent data, and not only on the original—the training—data on which it was developed. To this end, we ideally estimate the measures described above on independent test data.

To get a less biased estimate of the performance statistics in the training data, either cross-validation or bootstrapping is generally recommended. Bootstrapping is already in-built in some of the methodological approaches as described below. However, if feature selection is combined with model building, one needs to be aware that either a two-loop cross-validation or bootstrapping needs to be used. This means that a bootstrap sample is drawn in the first step. In the second step, the algorithm is trained and tuned on the in-bag samples. In the final step, the performance of the algorithm is evaluated using the out-of-bag samples. If model building and estimation is done on the same dataset, goodness of fit of the classification or prediction model may be substantially overestimated (Simon et al. 2003); for a discussion of different cross-validation approaches, see Molinaro et al. (2005).

Bootstrap and cross-validation can also be used to compare different algorithms on the training data; see, e.g., Malley et al. (2012). If test data and even different kinds of test data are available, the methods described by König et al. (2008) can be used for formal statistical comparisons of different machines.

It is important to note that bootstrapping and cross-validation are also often used for judging the stability of a model. However, validation is different from model stability. Specifically, even if variables appear in different bootstrap steps in very similar ways, this does not mean that using the same algorithm on independent data will give a similar model.

### What are typical results?

Although for many complex diseases, there have been impressive numbers of genetic regions identified to be associated, the typical results for classification and probability estimation are that the predictive values are only moderate (Gail 2008; Kooperberg et al. 2010). Many examples for this have been given by Janssens and van Duijn (2008), and one systematic collation of evidence on genetic tests is given by the Evaluation of Genomic Applications in Practice and Prevention (EGAPP) initiative (Teutsch et al. 2009). Some authors have argued that usually, too few markers have been included in the rule, which is substantiated in experiments (Evans et al. 2009; Hua et al. 2005a, b; Kooperberg et al. 2010; Raudys and Pikelis 1980; Wei et al. 2009; Zollanvari et al. 2011). Another reason might be that the way SNPs have been selected and combined is not well suited for the purpose of classification or probability estimation. As described above, SNPs are selected based on their strength of association with the phenotype. Again, this does not mean that they render good classification or probability estimation results. In addition, the combination of SNPs in scores is usually based on parametric regression models, which does not necessarily provide an optimal classification.

Therefore, it might be more meaningful to develop classification and probability estimation models using methods specifically targeted at classification and probability estimation. Specifically, machine-learning algorithms offer some advantages as described below. In consequence, there has been a rising trend to apply them also in the context of GWA data. To obtain an overview about what is possible and has been done in the GWA context, we will next provide a systematic review before we describe some of the methods in more detail.

## A systematic literature review on machine-learning approaches in the context of GWA studies

The aim of the systematic literature review was to gain an overview over which approaches have been used in the context of GWA data. For this purpose, we restricted the search to papers describing analyses of many SNPs, optimally from GWA studies, in humans. Other genetic variations such as microsatellites, copy number variations or gene expression levels were not considered. On the methods side, we considered supervised learning approaches only, although unsupervised methods may be used for the novel classification of subtypes of disease. An example for this is the genetic classification of Crohn’s disease subtypes (Cleynen et al. 2010).

**Fig. 2:**
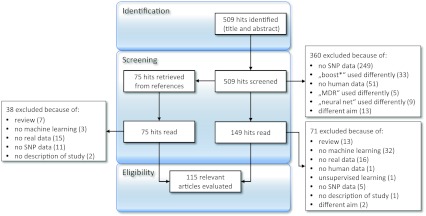
Flowchart of the systematic literature search

In detail, we started out by searching the PubMed database at http://www.ncbi.nlm.nih.gov/sites/entrez?db=PubMed on 1 September 2011, using the search terms shown in Table 1 and limiting the languages to English and German. This yielded 509 hits without duplicates. Based on titles and abstracts, we excluded 360 hits as shown in Fig. 2. The remaining 149 articles were read and a further 71 were excluded. The remaining 78 articles were evaluated, and their reference lists were screened for further relevant references. Additionally, hits identified as reviews were screened for further references. From these, another 75 articles were retrieved and read, and 38 excluded as shown in Fig. 2. Thus, 37 relevant articles were identified and evaluated.

**Table 1:** Results from PubMed search at ncbi.nlm.nih.gov/sites/entrez?db = PubMed on 1 September 2011

Search term	No. of hits
Genome-wide association machine learning	41
Genome-wide association random forest	15
Genome-wide association support vector	55
Genome-wide association boost*	24
Genome-wide association neural network	10
Genome-wide association logic regression	2
Genome-wide association MDR	15
SNPs machine learning	120
SNPs random forest	35
SNPs support vector	246
SNPs boost*	37
SNPs neural network	51
SNPs logic regression	21

* *Asterisk* indicates that the search is automatically expanded to all terms starting with this term

Of the identified 115 relevant articles in total, 91 described the application of machine-learning methods to SNPs in candidate genes or regions only, where these were defined based on previous results or biological knowledge. The number of SNPs analyzed per study ranged from 2 to 7,078 with a median of 39 SNPs per study. In 11 papers (Arshadi et al. 2009; Cleynen et al. 2010; Cosgun et al. 2011; Davies et al. 2010; Liu et al. 2011; Okser et al. 2010; Roshan et al. 2011; Wei et al. 2009; Yao et al. 2009; Zhang et al. 2010; Zhou and Wang 2007), SNPs were selected from a GWA study based on their marginal effects in single SNP association tests. In four of these papers (Arshadi et al. 2009; Liu et al. 2011; Roshan et al. 2011; Yao et al. 2009), the number of SNPs utilized exceeded 10 K. Two articles described the analysis of entire chromosomes with machine-learning methods (Phuong et al. 2005; Schwarz et al. 2009). Finally, 11 papers described the application of machine-learning methods to entire GWA data sets. Of these, two focused on the description of the method or software without a description of the results (Besenbacher et al. 2009; Dinu et al. 2007), and the remaining nine (Goldstein et al. 2010; Greene et al. 2010; Jiang et al. 2009, 2010; Schwarz et al. 2010; Wan et al. 2009; Wang et al. 2009; Wooten et al. 2010; Yang et al. 2011) are described in the following.

Five of the studies applying machine-learning algorithms to GWA data used random forests (RF; Goldstein et al. 2010; Jiang et al. 2009; Schwarz et al. 2010; Wang et al. 2009; Wooten et al. 2010) on a variety of disease phenotypes. Whereas Wooten et al. (2010) used RF to pre-select interesting SNPs based on their importance values, the others specified the aim as identification of associations (Goldstein et al. 2010; Wang et al. 2009) or gene–gene interactions (Jiang et al. 2009; Schwarz et al. 2010). Compared with the results from the previous classical analyses, all papers describe that novel genetic regions were identified but not yet validated.

In two further studies, multifactor dimensionality reduction (MDR, Moore 2010) was applied to detect gene–gene interactions in sporadic amyotrophic lateral sclerosis (Greene et al. 2010) and age-dependent macular degeneration (Yang et al. 2011). Based on this, Greene et al. (2010) developed a two-SNP classifier that was subsequently validated, and Yang et al. (2011) describe their results to be consistent with the original publications.

Wan et al. (2009) describe the development of a novel approach called MegaSNPHunter and applied it to Parkinson’s disease and rheumatoid arthritis. Again, they identified novel interactions that warrant independent validation. Finally, a Bayesian network approach was suggested by Jiang et al. (2010) and applied to the analysis of late-onset Alzheimer’s disease. Their results were in support of the original results, and interactions were not specifically looked at.

In summary, there were only very few applications of machine-learning methods to GWA data. Most of them supported classical results and named novel regions, which yet need to be validated in independent studies. Thus, the final success of these approaches cannot be judged at this time point.

A critical issue is that in no study, quality control was discussed in detail, but only standard control was applied. Given that most of the studies used publicly available data, this comes as no surprise. However, experience has shown that an ultimate quality control includes the visual inspection of the signal intensity plots (Ziegler 2009) which is still challenging to perform in a standardized way (Schillert et al. 2009).

A final point to note is that there was often obscurity about the use of terms in interpretations. Specifically, many papers seemingly aimed at the identification of interactions, but merely analyzed single SNP associations or classifications. Also, there was rarely a clear differentiation between classification or probability estimation and association as described above. Thus, we conclude that the real advantages of machine-learning approaches were not fully exhausted in most previous applications.

## Machine-learning approaches for classification and probability estimation

### Machine-learning approaches

Probability estimation and classification based on classical statistical approaches have not been vastly successful so far, and it might be more promising to use machine-learning approaches instead. Most machine-learning approaches are immanently built to render good classification, and only a few have been adapted to probability estimation (Malley et al. 2012). None of the machine-learning approaches are meant to statistically test for association.

Popular machine-learning approaches have been described in detail in some excellent textbooks and review papers. Table 2 lists the most popular approaches and provides references to the literature. In the “Appendix”, we describe classification and regression trees (CART), probability estimation trees (PETs), and RF for both classification (RF-Class) and probability estimation (RF-Reg) in more detail.

**Table 2:** Machine-learning approaches

Machine	Reference
Single machines	
Artificial neural networks (ANN)	Arminger and Enache (1996); Sarle (1994); Zou et al. (2008)
Diagonal linear discriminant analysis (DLDA)	Guo et al. (2007); McLachlan (2004)
*k*-nearest neighbors (*k*NN)	Steinbach and Tan (2009)
Linear discriminant analysis (LDA)	Guo et al. (2007); McLachlan (2004)
Logic regression	Chen et al. (2011); Schwender and Ruczinski (2010)
Logistic regression (logReg)	Hilbe (2009); Kleinbaum and Klein (2010)
Naïve Bayes	Hand (2009)
Quadratic discriminant analysis (QDA)	Guo et al. (2007); McLachlan (2004)
Support vector machines (SVM)	König et al. (2008); Noble (2006); Schölkopf and Smola (2002)
Tree-based methods:	Breiman et al. (1984)
C4.5	Ramakrishnan (2009)
Classification trees	Steinberg (2009)
Logistic regression tree with unbiased selection (LOTUS)	Chan and Loh (2004); Loh (2011)
CRUISE, M5, QUEST	Loh (2011)
Probability estimation trees (PETs)	Provost and Domingos (2003); Steinberg (2009)
Regression trees	Steinberg (2009)
Ensemble machines	
Boosting	Hastie et al. (2009); König et al. (2008)
Bootstrap aggregation (bagging)	Breiman (1996); König et al. (2008)
Deterministic forest	Zhang et al. (2003)
Random forest (RF)	Breiman (2001); König et al. (2008); Malley et al. (2012); Schwarz et al. (2010)

It is important to repeat that the classical logistic regression model or its generalizations rely on several crucial assumptions which are rather strict and limit the use of logistic regression in practice. In fact, to avoid problems in parameter estimation in case of misspecification, all important variables and their interactions must be correctly specified. A solution of this general probability estimation problem is obtained by treating it as a nonparametric regression problem. Informally, the aim is to estimate the conditional probability $\eta \left( {\user2{x}} \right) = {\mathbb{P}}\left( {y = 1\left| {\user2{x}} \right.} \right)$ of an observation *y* being equal to 1 given the variables *x*. By noting that ${\mathbb{P}}\left( {y = 1\left| {\user2{x}} \right.} \right) = {\mathbb{E}}\left( {y\left| {\user2{x}} \right.} \right)$, it can be seen that the probability estimation problem is identical to the nonparametric regression estimation problem $f\left( {\user2{x}} \right) = {\mathbb{E}}\left( {y\left| {\user2{x}} \right.} \right)$. Hence, any learning machine performing well on the nonparametric regression problem $f\left( {\user2{x}} \right)$ will also perform well on the probability estimation problem $\eta \left( {\user2{x}} \right)$.

The nonparametric regression estimation problem has been considered in the literature in detail (Devroye et al. 1996; Györfi et al. 2002), and many learning machines are already available. These include RF, *k*-nearest neighbors, kernel methods, artificial neural networks or bagged *k*-nearest neighbors. However, some learning machines are known to be problematic and may not allow consistent estimation of probabilities (Malley et al. 2012; Mease and Wyner 2008; Mease et al. 2007). Large-margin support vector machine (SVM) classifiers can also be used for consistent probability estimation (Wang et al. 2008). There are, however, conceptual differences in the probability estimation approaches for those SVM machine-learning approaches which have generally been proven to provide consistent estimates (for a discussion, see Malley et al. 2011).

### Consistency of probability estimates

The reader needs to be aware that some software packages seem to offer probability estimation using specific options, such as the prob option in the randomForest package of R. However, the availability of such an option does not mean that its output may be interpreted as a consistent estimate of a probability. Consistency means that the estimate of the probability converges to its true probability value if the sample size tends to infinity.

Some machines are not universally consistent. For example, even RF is not consistent if splits are performed to purity. Thus, if trees are grown to purity so that only a single observation resides in a terminal node, the probability estimate is based on only a sample of size *n* = 1. Averaging over a number of trees in the corresponding RF does not necessarily generate correct probabilities. Therefore, some impurity within the tree is required for consistency of RF. In contrast, bagging over trees split to purity does return consistency (Biau et al. 2008). In addition, bagged nearest neighbors provide consistent probability estimates under very general conditions (Biau and Devroye 2010; Biau et al. 2008). For the consistency of artificial neural networks and kernel methods, the reader may refer to Györfi et al. (2002, Ch. 6). The reader should, however, note that neural networks belong to the class of model-based approaches, and the relationship between neural networks and regression analysis has been well established (Sarle 1994).

The final question is whether consistent probability estimates can be obtained under any sampling scheme. The simple answer to this question is no. In fact, prospective sampling, not case–control or cross-sectional sampling, is required to guarantee unbiased probability estimates. This has been considered in detail for the logistic regression model by Prentice and Pyke (1979) and by Anderson (1972). If the logistic regression model is applied to data from a case–control study, the regression coefficients are identical. Only the estimate of the intercept is different. More specifically, the intercept α of the prospective likelihood is a simple function of the intercept of the retrospective likelihood α*, and it is given by α = α* + ln(π_1_/π_0_), where π_1_ and π_0_ are the sampling proportions of cases and controls, respectively, from the general population. Thus, if the sampling proportions are known, probabilities can be estimated as if the data came from a prospective study.

A similar function for relating prospective and retrospective study designs is unknown for machine-learning approaches. Thus, the interpretation of probability estimates from machine-learning approaches based on retrospective data is not necessarily consistent.

## Examples for data analysis: genome-wide association data on rheumatoid arthritis

### Description and preparation of the data

To illustrate some of the methods described so far, we applied them to a data set from a GWA study on rheumatoid arthritis. This data set had been provided for the Genetic Analysis Workshop 16 (Amos et al. 2009) and comprises 868 cases and 1,194 controls who had been genotyped on the Illumina 550k platform.

After exclusion of monomorphic SNPs and SNPs showing deviation from Hardy–Weinberg equilibrium at *p* < 0.0001, 515,680 SNPs were available for further analysis. Population stratification is known to be prevalent in this data set (Hinrichs et al. 2009), and we accordingly estimated the inflation factor λ to be 1.39. Therefore, we used multidimensional scaling with pruned SNPs to obtain an unstratified subset of individuals. Exclusion of 617 subjects reduced λ to 1.05 using the pruned SNPs. Further analyses were thus based on 707 cases and 738 controls.

Missing genotypes were imputed using PLINK (version 1.07, Purcell et al. 2007) with default method and parameters. The entire HapMap (release 23, 270 individuals, 3.96 million SNPs) was utilized as reference panel for the imputation. A negligible number of SNPs could not be imputed, resulting in 506,665 SNPs with complete data for further analysis.

To obtain independent data sets for rule construction and rule evaluation, the data set was split into a training (476 cases and 487 controls) and a test data set (231 cases and 251 controls).

### Construction of classification and probability estimation rules

In the training data set, we performed single SNP analyses using a trend test resulting in associations shown in Supplementary Fig. 1. Based on a genome-wide significance threshold of 5 × 10^−8^, 183 SNPs were associated with disease status. Analyzed in the test data set, 65 SNPs of these were again genome-wide significant.

To construct classification and risk scores in the training data, we used the following approaches:

- “allele count”: count the number of risk alleles over all included SNPs for every person,
- “logOR”: weight SNPs using respective log odds ratio from single SNP analysis,
- “lasso”: least absolute shrinkage and selection operator (lasso) combining shrinkage of variable parameter estimates with simultaneous variable selection by shrinking some of the coefficients of the full model to zero (Tibshirani 1996); extent of shrinkage was determined using tenfold cross-validation to identify the parameter with highest cross-validated classification accuracy,
- “logReg”: logistic regression model using the SNPs in the smallest set (see below) simultaneously,
- “RJ-Reg”: RFs in the regression mode using Random Jungle (Schwarz et al. 2010); default parameters for probability estimation were used with stopping at a terminal node size of five to get consistent probability estimators.

It should be noted that only the logReg, the lasso and the RJ-Reg methods render probability estimates as scores, whereas the logOR and the allele count method yield continuous scores.

To vary the number of SNPs used in a specific score, we performed a backstep iteration procedure within the RF approach. Starting with the complete set of SNPs and then within every iteration, the Liaw score was computed. Then, the 50 % more important SNPs were kept iteratively for the next step yielding successively smaller SNP sets. From these, we selected eight different sets with the number of SNPs ranging between 63 (0.012 %) and 63,334 (12.5 %), where the last set was only used for the logOR and the RJ-Reg method.

For a binary classification, we selected the threshold that maximized the Youden index in the training data for the scores based on allele count, logOR, logReg and lasso. For RFs, Random Jungle was utilized in the classification mode, again using default parameters but without pruning. The resulting classification is termed “RJ-Class”.

### Evaluation of classification and probability estimation rules

**Fig. 3:**
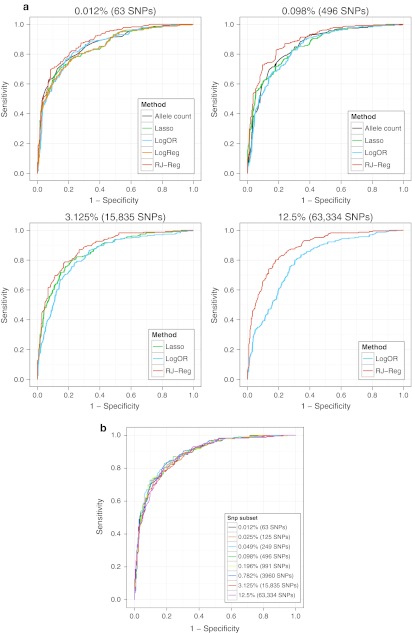
**a** ROC curves for all methods in selected SNP sets in the test data. **b** ROC curves for Random Jungle in regression mode in all SNP sets in the test data

Every score applied to the training and test data was evaluated in the test data by plotting ROC curves (Fig. 3a showing methods across selected SNP sets and Fig. 3b showing different SNP sets for RJ-Reg) and estimating AUCs with 95 % CIs (Table 3). We compared the AUCs within one methodological approach as well as within one SNP set using the method by DeLong et al. (1988). The detailed comparison results are given in Supplementary Table 1.

**Table 3:** Areas under the curve for all scores in the training and test data

SNP selection	Score	AUC train (95 % CI)	AUC test (95 % CI)
0.012 %	Allele count	0.9075 (0.8898; 0.9252)	0.8644 (0.8320; 0.8968)
(63 SNPs)	LogOR	0.8824 (0.8617; 0.9030)	0.8565 (0.823; 0.8900)
	LogReg	0.9449 (0.9321; 0.9577)	0.8492 (0.8152; 0.8831)
	Lasso	0.9433 (0.9303; 0.9563)	0.8511 (0.8174; 0.8849)
	RJ-Reg	1.0000 (0.9999; 1.0000)	0.8883 (0.8599; 0.9167)
0.025 %	Allele count	0.8964 (0.8770; 0.9158)	0.8527 (0.8189; 0.8866)
(125 SNPs)	LogOR	0.8602 (0.8373; 0.8832)	0.8326 (0.7966; 0.8686)
	Lasso	0.9573 (0.9464; 0.9683)	0.8604 (0.8279; 0.8928)
	RJ-Reg	1.0000 (0.9999; 1.0000)	0.8877 (0.8591; 0.9163)
0.049 %	Allele count	0.9288 (0.9132; 0.9444)	0.8510 (0.8168; 0.8852)
(249 SNPs)	LogOR	0.8733 (0.8515; 0.8950)	0.8374 (0.8019; 0.8729)
	Lasso	0.9824 (0.9763; 0.9885)	0.8622 (0.8298; 0.8945)
	RJ-Reg	1.0000 (1.0000; 1.0000)	0.8925 (0.8644; 0.9206)
0.098 %	Allele count	0.9548 (0.9436; 0.9660)	0.8565 (0.8230; 0.8900)
(496 SNPs)	LogOR	0.8884 (0.8682; 0.9085)	0.8426 (0.8076; 0.8775)
	Lasso	0.9960 (0.9939; 0.9981)	0.8555 (0.8228; 0.8882)
	RJ-Reg	1.0000 (1.0000; 1.0000)	0.8914 (0.8631; 0.9198)
0.196 %	Allele count	0.9742 (0.9659; 0.9824)	0.8248 (0.7881; 0.8615)
(991 SNPs)	LogOR	0.9092 (0.8913; 0.9271)	0.8429 (0.8080; 0.8778)
	Lasso	0.9987 (0.9979; 0.9996)	0.8495 (0.8155; 0.8834)
	RJ-Reg	1.0000 (1.0000; 1.0000)	0.8902 (0.8617; 0.9188)
0.782 %	Allele count	0.9075 (0.8898; 0.9252)	0.7251 (0.6803; 0.7700)
(3960 SNPs)	LogOR	0.9616 (0.9513; 0.9719)	0.8456 (0.8110; 0.8802)
	Lasso	1.0000 (1.0000; 1.0000)	0.8477 (0.8136; 0.8817)
	RJ-Reg	1.0000 (1.0000; 1.0000)	0.8919 (0.8634; 0.9203)
3.125 %	Allele count	0.9967 (0.9950; 0.9984)	0.6474 (0.5988; 0.6961)
(15,835 SNPs)	LogOR	0.9982 (0.9970; 0.9982)	0.8340 (0.7977; 0.8340)
	Lasso	1.0000 (0.9999–1.0000)	0.8586 (0.8257; 0.8916)
	RJ-Reg	1.0000 (1.0000; 1.0000)	0.8829 (0.8534; 0.9124)
12.5 %	LogOR	1.0000 (1.0000; 1.0000)	0.7984 (0.7590; 0.8378)
(63,334 SNPs)	RJ-Reg	1.0000 (1.0000; 1.0000)	0.8854 (0.8563; 0.9146)

*AUC* area under the curve, *CI* confidence interval, *Lasso* least absolute shrinkage and selection operator, *RJ-Reg* Random Jungle regression

Allele count: score constructed based on number of risk alleles

LogOR: score constructed by weighting variants with respective log odds ratio from single marker analyses

LogReg: score constructed from logistic regression

Within the allele count method, we found that smaller SNP sets yielded higher AUCs. The pattern was more irregular for the logOR method; here, AUC was lowest for the 0.025 and 0.049 % as well as for the 12.5 % SNP set. No differences in AUC were observed for the lasso method. Finally, for RJ-Reg, AUC was highest for medium SNP sets with 0.049 to 0.782 % of the total number of SNPs.

On comparing the methods within one SNP set, we found that overall, RJ-Reg led to higher AUCs than any of the other methods in any SNP set. Furthermore, the allele count method rendered a higher AUC than the logOR method in the 0.025 % and the 0.049 % SNP sets, but was worse than the lasso or the logOR method within the 0.782 % SNP set.

**Fig. 4:**
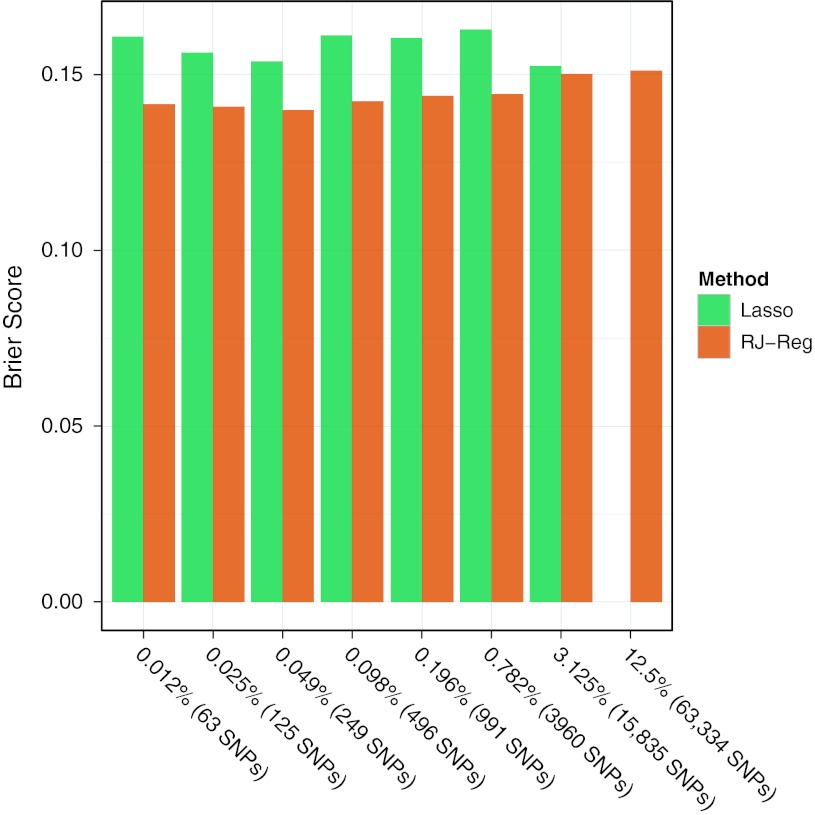
Brier scores for scores based on lasso or Random Jungle regression in the test data

We estimated the Brier score that is based on the squared differences between observed and predicted probabilities. As this requires estimated probabilities, we could only use this for the methods lasso and RJ-Reg, and the results are shown in Fig. 4. It should be noted that this analysis is for illustration only, since the comparison of probabilities usually requires risk estimates from a prospective study design.

For a binary classification, for every score, we selected the threshold that maximized the Youden index in the training data. Then, sensitivity and specificity were calculated with 95 % CIs according to Wilson (1927) and are shown in Table 4. For a direct comparison between methods and SNP sets in the test data, we calculated the differences in the proportions of correctly classified subjects with 95 % CIs using the method by Zhou and Qin (2005).

**Table 4:** Sensitivity and specificity for all scores in the training and test data

SNP selection	Score	Sens train (95 % CI)	Spec train (95 % CI)	Sens test (95 % CI)	Spec test (95 % CI)
0.012 %	Allele count	0.8256 (0.7890; 0.8571)	0.8255 (0.7892; 0.8566)	0.7532 (0.6938; 0.8044)	0.8167 (0.7642; 0.8597)
(63 SNPs)	LogOR	0.8025 (0.7644; 0.8358)	0.8029 (0.7652; 0.8358)	0.7489 (0.6892; 0.8005)	0.8247 (0.7729; 0.8667)
	LogReg	0.8655 (0.8320; 0.8933)	0.8645 (0.8312; 0.8920)	0.7489 (0.6892; 0.8005)	0.7928 (0.7385; 0.8384)
	Lasso	0.8676 (0.8342; 0.8952)	0.8686 (0.8357; 0.8957)	0.7403 (0.6801; 0.7925)	0.8008 (0.7470; 0.8455)
	RJ-Class	1.0000 (0.9920; 1.0000)	1.0000 (0.9922; 1.0000)	0.7706 (0.7122; 0.8201)	0.8207 (0.7685; 0.8632)
0.025 %	Allele count	0.8130 (0.7755; 0.8455)	0.8070 (0.7696; 0.8396)	0.7489 (0.6892; 0.8005)	0.8088 (0.7556; 0.8526)
(125 SNPs)	LogOR	0.7689 (0.7290; 0.8045)	0.7700 (0.7306; 0.8052)	0.7143 (0.6529; 0.7687)	0.7610 (0.7045; 0.8095)
	Lasso	0.8866 (0.8549; 0.9120)	0.8871 (0.8559; 0.9122)	0.7576 (0.6984; 0.8083)	0.8088 (0.7556; 0.8526)
	RJ-Class	1.0000 (0.9920; 1.0000)	1.0000 (0.9922; 1.0000)	0.7662 (0.7076; 0.8162)	0.8207 (0.7685; 0.8632)
0.049 %	Allele count	0.8529 (0.8183; 0.8819)	0.8583 (0.8245; 0.8865)	0.7532 (0.6938; 0.8044)	0.7968 (0.7427; 0.8419)
(249 SNPs)	LogOR	0.7773 (0.7378; 0.8124)	0.7782 (0.7392; 0.8129)	0.7273 (0.6665; 0.7806)	0.7610 (0.7045; 0.8095)
	Lasso	0.9328 (0.9066; 0.9520)	0.9322 (0.9064; 0.9513)	0.7532 (0.6938; 0.8044)	0.7968 (0.7427; 0.8419)
	RJ-Class	1.0000 (0.9920; 1.0000)	1.0000 (0.9922; 1.0000)	0.7922 (0.7353; 0.8395)	0.8088 (0.7556; 0.8526)
0.098 %	Allele count	0.8782 (0.8457; 0.9045)	0.8665 (0.8334; 0.8939)	0.7359 (0.6756; 0.7886)	0.8207 (0.7685; 0.8632)
(496 SNPs)	LogOR	0.7983 (0.7599; 0.8319)	0.7967 (0.7587; 0.8301)	0.7316 (0.6710; 0.7846)	0.7649 (0.7087; 0.8132)
	Lasso	0.9622 (0.9410; 0.9759)	0.9671 (0.9473; 0.9797)	0.7056 (0.6439; 0.7607)	0.8207 (0.7685; 0.8632)
	RJ-Class	1.0000 (0.9920; 1.0000)	1.0000 (0.9922; 1.0000)	0.8009 (0.7446; 0.8473)	0.8048 (0.7513; 0.8491)
0.196 %	Allele count	0.9223 (0.8947; 0.9431)	0.9138 (0.8855; 0.9356)	0.7143 (0.6529; 0.7687)	0.7849 (0.7299; 0.8312)
(991 SNPs)	LogOR	0.8256 (0.7890; 0.8571)	0.8255 (0.7892; 0.8566)	0.7316 (0.6710; 0.7846)	0.7849 (0.7299; 0.8312)
	Lasso	0.9790 (0.9618; 0.9885)	0.9795 (0.9626; 0.9888)	0.7056 (0.6439; 0.7607)	0.8406 (0.7903; 0.8807)
	RJ-Class	1.0000 (0.9920; 1.0000)	1.0000 (0.9922; 1.0000)	0.7965 (0.7400; 0.8434)	0.7809 (0.7257; 0.8276)
0.782 %	Allele count	0.9370 (0.9115; 0.9555)	0.9363 (0.9111; 0.9548)	0.6061 (0.5418; 0.6668)	0.7092 (0.6502; 0.7619)
(3,960 SNPs)	LogOR	0.8971 (0.8665; 0.9213)	0.8973 (0.8672; 0.9213)	0.7143 (0.6529; 0.7687)	0.8127 (0.7599; 0.8562)
	Lasso	1.0000 (0.9920; 1.0000)	1.0000 (0.9922; 1.0000)	0.6926 (0.6304; 0.7486)	0.8327 (0.7816; 0.8738)
	RJ-Class	1.0000 (0.9920; 1.0000)	1.0000 (0.9922; 1.0000)	0.7792 (0.7214; 0.8279)	0.7610 (0.7045; 0.8095)
3.125 %	Allele count	0.9685 (0.9487; 0.9808)	0.9671 (0.9473; 0.9797)	0.5455 (0.4810; 6084)	0.6175 (0.5561; 0.6755)
(15,835 SNPs)	LogOR	0.9832 (0.9672; 0.9915)	0.9836 (0.9679; 0.9917)	0.7576 (0.6984; 0.8083)	0.7689 (0.7130; 0.8168)
	Lasso	1.0000 (0.9920; 1.0000)	1.0000 (0.9922; 1.0000)	0.7792 (0.7214; 0.8279)	0.7928 (0.7385; 0.8384)
	RJ-Class	1.0000 (0.9920; 1.0000)	1.0000 (0.9922; 1.0000)	0.7532 (0.6938; 0.8044)	0.7649 (0.7087; 0.8132)
12.5 %	LogOR	1.0000 (0.9920; 1.0000)	1.0000 (0.9922; 1.0000)	0.6883 (0.6259; 0.7446)	0.7490 (0.6919; 0.7986)
(63,334 SNPs)	RJ-Class	1.0000 (0.9920; 1.0000)	1.0000 (0.9922; 1.0000)	0.7446 (0.6847; 0.7965)	0.7769 (0.7214; 0.8240)

*Sens* sensitivity, *CI* confidence interval, *spec* specificity, *Lasso* least absolute shrinkage and selection operator, *RJ-Class* Random Jungle classification

Allele count: score constructed based on number of risk alleles

Log OR: score constructed by weighting variants with respective log odds ratio from single marker analyses

LogReg: score constructed from logistic regression

The detailed results in Supplementary Table 1 show that these analyses mostly mirror the results from comparing the AUCs. The only remarkable difference was that for RJ-Class, smaller SNP sets led to a better classification, although for RJ-Reg, medium SNP sets had shown the best AUC.

In summary, the prediction accuracy based on continuous scores or probabilities was usually better when using RJ-Reg as compared to the other methods. The number of SNPs for an optimal prediction was dependent on the method, whereas it played no role when using the lasso. Smaller SNP sets were better for the allele count method, but a medium number of SNPs was optimal for the RJ-Reg.

## Conclusions

Although based on one small data set, our analysis of a GWA study on rheumatoid arthritis showed two things. Firstly, when different SNP sets were compared, our results did not substantiate previous results that using more SNPs yielded better results; instead, our results indicated that the best SNP set may depend on the actual method used for rule construction. Secondly, in this data set, there was a consistent advantage of using Random Jungle over other methods.

In contrast, our literature review showed that machine-learning algorithms have so far been underutilized. Moreover, when applied, their specific value with regard to classification and probability estimation has usually not been exhausted.

In line with this, we make a plea for clearer definitions of the terms and study aims. Specifically, association, classification and probability estimation can be different aims of studies, require different methods, and result in different interpretations.

### Electronic supplementary material

Below is the link to the electronic supplementary material.

Supplementary material 1 (PPTX 57 kb) Manhattan plot showing −log *p* values from single SNP trend tests of association with rheumatoid arthritis

Supplementary material 2 (XLSX 23 kb) Comparison of the area under the curves (AUCs) and percentage of correct classifications across methods and SNP sets

## Appendix

### Classification and regression trees, probability estimation trees, and random forests for classification and probability estimation

The overall goal of CART is to generate a decision tree that classifies individuals correctly (Breiman et al. 1984). The objective in the partitioning thus is to identify subgroups of individuals who are increasingly homogeneous with respect to their outcome. The overall goal of PET is similar, but a decision tree is generated for estimating the response probabilities.

Beginning at the root node with the entire sample of patients, one follows the stem to its branches. At each node of the tree, the sample is split, until, in the last branches, the subset of patients is relatively homogeneous. Details of the CART algorithm are described, e.g., in König et al. (2008) or Steinberg (2009), and the PET procedure is almost identical. Here, we sketch the CART procedure.

Beginning with the entire data as the first node, the feature space is partitioned into two branches. These in turn become the nodes for the next partitioning. Trees are grown to their maximal size and no stopping rule is applied. Tree growing thus stops when no further splits are possible because of lack of data. The maximal tree is then pruned back to the root using the split with the least contribution to the overall performance of the tree for pruning. In the final step, the optimal tree is selected.

The final size of the trees is an important parameter in the tree-growing process. The larger the tree, the more difficult the results are to interpret. Smaller trees are easier to understand, but they might not adequately reflect complex data structures. Thus, larger trees exploit more of the available information for accurate classifications, but tend to overfit the data. Subsequently, there is loss in generalization to new data. For PETs, the situation is worse and the node probability estimates form a single tree can be very misleading, irrespective of the tree size (Provost and Domingos 2003; Steinberg 2009).

For the analysis of high-dimensional data, tree growing to purity with subsequent pruning and tree selection is not computer efficient. Algorithms not growing the tree to purity, not using pruning and optimal tree selection would be preferable. For computational speed-up, the growing process might therefore be stopped when (Carayol et al. 2010; Malley et al. 2012):

1. only cases with the same outcome remain in every child node,
2. all cases within every child node have identical predictor variables,
3. an external limit on the depth or the complexity of the tree has been reached,
4. the node size is just above or below a pre-defined threshold, such as 5 or 10 % of all samples.

Two additional aspects of the tree-growing process are important for the following considerations.

First, CART aims at maximizing the average purity of the two child nodes in the partitioning step. Different measures of purity, i.e., splitting criteria can be applied. While the mean square error is generally used as splitting criterion for regression trees, the misclassification error or the Gini index is typically used. For classification trees, we generally prefer the Gini index because of its functional relation to the variance (Carayol et al. 2010), and for probability estimation we use the mean square error (Malley et al. 2012) as in regression trees.

Second, both CART and PET can be done using a single tree. A new subject is dropped down the tree to its terminal node, also termed leaf node. For classification, the new subject is assigned the status of the majority of the subjects residing in the terminal node. For example, if six cases and two controls are in the terminal node of the new subject, the majority vote says that the new subject gets a case assignment. For probability estimation, the proportion of cases divided by the total sample size is determined and used as estimate. In the example, the new subject is a case with a probability of 6/8 = 75 %. This approach traces back to Breiman et al. (1984, Sect. 5.4), but PETs generally produce poor estimates of class probabilities (Provost and Domingos 2003).

Although the procedure of growing trees is intuitive, there are some disadvantages to CART and PET, and these include the problem that the resulting trees have a high variance. This means that small changes in the data can result in extremely different trees, thus different interpretations, distinct predictions for individual cases and widely varying error fractions. Furthermore, PETs yield biased probability estimates.

The use of an ensemble of trees by creating a forest generally leads to both improved classifications and probability estimates (Bauer and Kohavi 1999; Breiman 2001; Buntine 1992; Provost and Domingos 2003; Provost et al. 1998). In fact, it can be shown that probabilities can be estimated consistently from RF if some tree-building rules are met (Biau et al. 2008; Malley et al. 2012); see “Machine-learning approaches for classification and probability estimation”.

We now describe the basic RF algorithm. As in Breiman (2001), consider a training data set drawn from a sample of independently identically distributed random variables, where each subject *i* is a pair of a feature vector $x_{i}$ and a dichotomous outcome $y_{i}$. A test subject is dropped down the tree in the usual RF manner and soon resides in a terminal node. Under classification in RF (*RF-Class*), a classification is made in each tree by taking a majority vote in this terminal node of the tree. Under regression in RF (*RF-Reg*), an estimate of the probability of *y* given the features *x* is obtained. This is done by averaging the estimated proportion of case observations in the training data set over all trees in the forest. We stress that the terms *RF-Class* and *RF-Reg* are not related to the split criteria used for generating the RF, although the split criterion might affect the performance of the RF. The general *RF-Reg* procedure takes the following steps (Malley et al. 2012):

1. Consider a training data set of size *n*.
2. A bootstrap sample *b* consisting of *n* samples drawn with replacement is drawn from the original training data set. The samples left out due to the bootstrapping process are called ‘out-of-bag’ (OOB) data.
3. A PET is grown using the bootstrap data set. For splitting data, all splits of a random subset of features are considered.
4. The PET is grown to the greatest extent possible but requiring a minimum nodesize of *k* % of the sample. In our applications, we tune the proportion of samples in the terminal node (unpublished). No pruning is performed.
5. The proportion of cases in each terminal node of the PET is determined.
6. Steps 2–5 are repeated to grow a specific number of trees, ntree.
7. To estimate the probability of a new subject, it is dropped down a tree until its final node. The proportion of cases in this final node is determined. The probability estimate is the proportion of cases averaged over all ntree trees.

For *RF-class*, only steps 3 and 5 in the algorithm are altered. Specifically, in 3 a dichotomous purity measure, such as the Gini index is used instead of the MSE (Schwarz et al. 2010). In step 5, the majority vote is taken in a terminal node. Step 4 of the algorithm is not standard because tree growing is stopped in some implementations when ≥5 observations are left in the terminal node, regardless of sample size, or they are grown to purity.

Several options are available with RFs, such as the estimation of variable importance measures (Nicodemus et al. 2010), the estimation of the most representative tree (Banerjee et al. 2012) or the calculation of proximities between subjects. For this, every subject is dropped down each tree, and each pair of subjects is compared with regard to the final stopping point. If they are classified into the same final node in a single tree of the forest, the proximity between them is increased by one. The resulting values can be used to replace missing data and to identify outliers.
