# Supplementary figures and images for: Risk estimation and risk prediction using machine-learning methods
Source: Hum Genet. 2012 Jul 3;131(10):1639–54. doi: 10.1007/s00439-012-1194-y (PMC3432206; doi:10.1007/s00439-012-1194-y)

## Slide 1
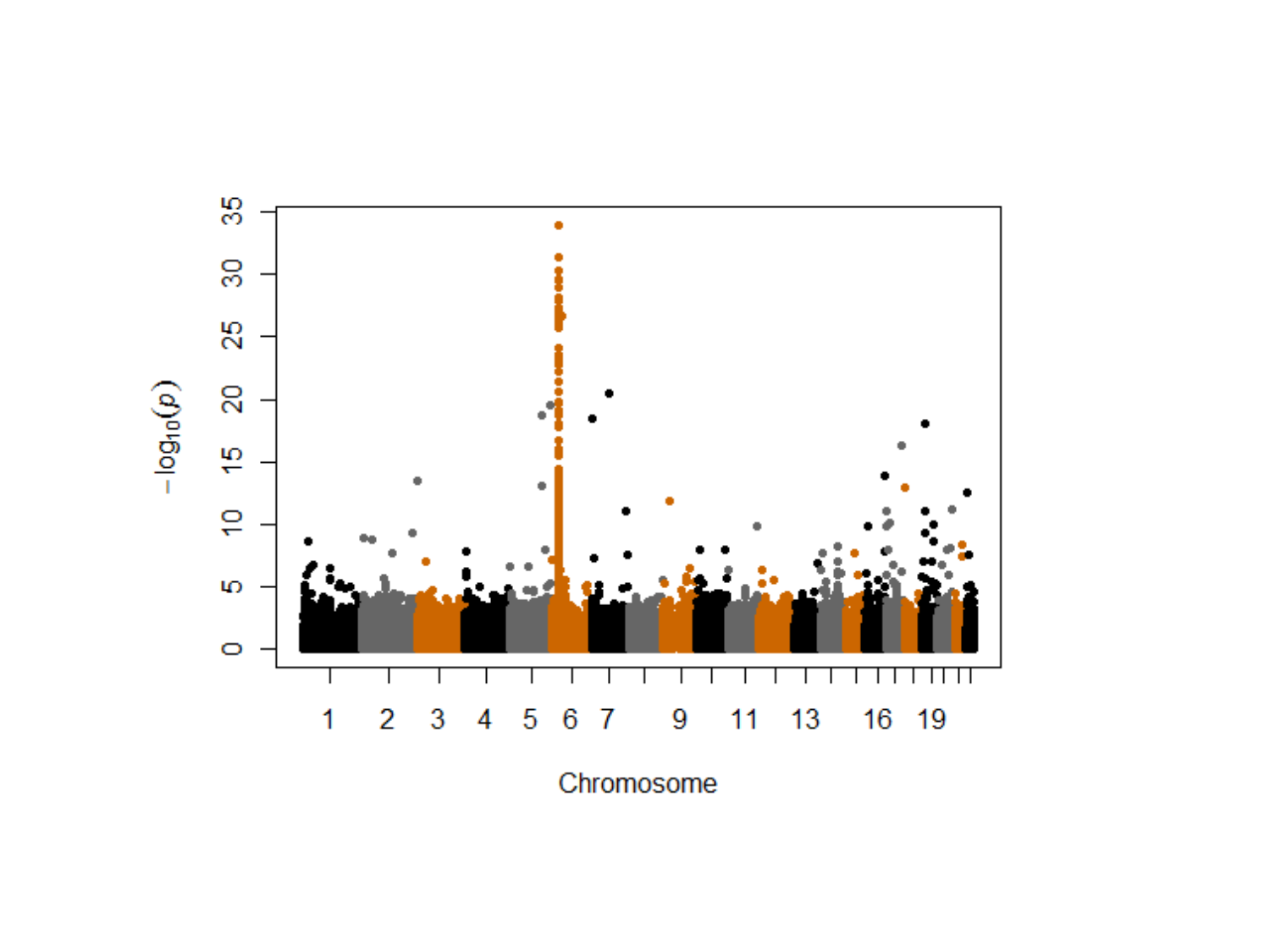

Supplement: Supplementary file 1 — Supplementary material 1 (PPTX 57 kb) Manhattan plot showing −log p values from single SNP trend tests of association with rheumatoid arthritis [file 439_2012_1194_MOESM1_ESM.pptx]
